# Social Media Validation and Self-Esteem: A Case Study on the Mediating Role of Social Comparison on Instagram

**DOI:** 10.1192/j.eurpsy.2026.11363

**Published:** 2026-07-31

**Authors:** A. Argyriadi, P. K. Kouassi, M. Liu, A. Argyriadis

**Affiliations:** 1 Psychology, American University in Dubai, Dubai, United Arab Emirates; 2 Nusrsing, Hellenic Mediterranean University, Heraklion Crete, Greece

## Abstract

**Introduction:**

Instagram remains one of the most widely used platforms among young adults, where validation is quantified through likes, comments, and followers. According to social comparison theory, upward and downward comparisons significantly shape self-perception and self-esteem. Understanding these dynamics in real-life contexts is crucial for designing preventive and psychoeducational interventions in university populations.

**Objectives:**

This case study aimed to explore how Instagram-based validation influences self-esteem through social comparison, and to identify protective factors that buffer potential negative outcomes.

**Methods:**

A multiple-case qualitative study across two universities with five course cohorts (N = 124, ages 18–25). Data sources over four weeks: (a) 8 focus groups (10–12 students each) on Instagram practices and perceived validation; (b) weekly reflective diaries mapping episodes of upward/downward comparison; (c) embedded scales (Rosenberg Self-Esteem; Iowa–Netherlands Comparison Orientation; brief Mindful Attention Awareness; Multidimensional Scale of Perceived Social Support) for descriptive context. Reflexive thematic analysis identified cross-case patterns; quantitative summaries (medians/IQRs; within-cohort correlations) contextualized themes.

**Results:**

Across cases, upward comparisons to peers/influencers consistently co-occurred with transient drops in self-evaluation and heightened self-consciousness around posting; downward comparisons yielded brief, weaker boosts. Students who de-emphasized metrics (hiding like counts, limited checking) and reported higher mindfulness/social support described attenuated reactivity to validation cues. Descriptive summaries aligned with themes (higher comparison orientation paired with lower self-esteem within cohorts).

**Conclusions:**

At the group case level, Instagram validation relates to self-esteem chiefly through social comparison dynamics, with mindfulness and social support acting as protective buffers. University mental-health programming that targets comparison habits, digital literacy, and mindful media use may strengthen resilience against validation-linked self-esteem fluctuations. Further mixed-methods and longitudinal case replications are recommended.

**Disclosure of Interest:**

None Declared

